# Prevalence of CYP2C19 Genetic Polymorphism among Normal People and Patients with Hepatic Diseases

**Published:** 2018-02-01

**Authors:** Z. Hashemizadeh, S. A. Malek-Hosseini, P. Badiee

**Affiliations:** 1 Prof. Alborzi Clinical Microbiology Research Center, Shiraz University of Medical Sciences, Shiraz, Iran; 2 Transplant Research Center, Shiraz University of Medical Sciences, Shiraz, Iran

**Keywords:** Cytochrome P450, CYP2C19, Hepatic disorders, Liver transplantation

## Abstract

**Background:**

Patients with hepatic diseases are treated with numerous drugs metabolized by cytochrome P450.

**Objective:**

To evaluate the frequencies of CYP2C19 variant alleles (*2, *3, and *17), genotypes, and phenotypes, and the relationship between the frequency of these alleles and the underlying hepatic diseases among patients with advanced liver diseases who were candidates for liver transplantation.

**Methods:**

The Study was conducted on 120 patients suffering from various hepatic disorders, candidates for liver transplantation, and 52 healthy volunteers. DNA was extracted from blood samples and analyzed by TaqMan SNP genotyping assay. The CYP2C19 genotypes were classified into poor, extensive, intermediate, and ultra-rapid metabolizer phenotypes.

**Results:**

Viral hepatitis was the most common cause of liver disease among studied patients. The frequencies of CYP2C19 alleles *1, *17, and *2 were 66.7% (160/240), 20.8% (50/240) and 12.5% (30/240), respectively. Allele CYP2C19*3 was not found in the studied population. The most prevalent genotypes were CYP2C19 *1/*1 (47.5%) and *1/*17 (24.2%). The predicted CYP2C19 phenotypes were extensive metabolizer (47.5%), heterozygote extensive metabolizer (45.9%), ultra-rapid metabolizer (5%), and poor metabolizer (1.6%). There was no significant difference between the frequencies of CYP2C19 genotypes between healthy people and patients. The distribution of CYP2C19 genotype frequencies was not significantly associated with the underlying disease conditions (p=0.472).

**Conclusion:**

The distribution of CYP2C19 genotype frequencies in Iranian healthy people and patients with various hepatic diseases was not significantly different. This may allow the physicians to predict a tailoring dose regimens based on the individual’s metabolic capacity, decrease the risk of harmful side effects of the drugs, and optimize the treatment.

## INTRODUCTION

Genetic polymorphism of drug-metabolizing enzymes is mostly associated with inter-individual and inter-ethnic variations in the pharmacokinetic and pharmacodynamic responses to drugs. This may influence the drug action and lead to differences in the efficacy or toxicity of pharmacological agents used [1]. Among several genes responsible for drug metabolism, cytochrome P450 genes have an important role in encoding many enzymes involved in the metabolism of a wide variety of drugs [2]. The hepatic cytochrome P450 2C19 (CYP2C19) is one of the main isoforms of cytochrome P450 2C subfamily, represented by related genes located on chromosome 10 [3, 4]. Many variant alleles have been identified for CYP2C19, as a highly polymorphic gene. The wild-type CYP2C19*1 allele is the completely functional form of the CYP2C19 identified [5]. The most frequent CYP2C19 loss-of-function polymorphic alleles are two defective variants including *2 (rs4244285) with single-base substitutions (681G>A), resulting in a splicing defect and *3 (rs4986893) with a point mutation (636G>A), producing a premature stop codon [6]. CYP2C19*2 and *3 are the variant alleles with insufficient activity that can cause null function of the enzyme [6]. The other CYP2C19 gene variant is *17 (rs-12248560), located in a single nucleotide polymorphism (SNP, 806C>T) in a transcriptional regulatory region of the gene, leading to the enhancement of the transcriptional activity of the enzyme and ultra-rapid metabolism of CYP2C19 substrates [7]. Various combinations of these alleles lead to different classes of CYP2C19 phenotypes: poor metabolizer (PM, CYP2C19*2*2 and *3*3), intermediate metabolizer (HEM, heterozygote extensive matabolizer, CYP2C19*1/*2, CYP2C19*1/*17, CYP2C19*2/*17), extensive metabolizer (EM, CYP2C19*1/*1), and ultra-rapid metabolizer (URM, CYP2C19*17*17) [6, 7]. Various studies have shown great ethnic differences in the frequency of PM phenotypes, ranging from 3% to 5% in Caucasians, 12% to 23% in Orientals [8], and 4% to 8% in Africans [9]. The frequency of URM was reported to be 18% to 26% in Caucasian [10], and 0.4% to 1.4% in Asian populations [11]. 

The efficacy and safety of pharmacogenetic drugs are related to the gene frequencies in different populations. Patients suffering from hepatic disorders and candidates for liver transplantation use numerous drugs, the metabolisms of which depend on the cytochrome P450 activity. The objective of this study was to evaluate the frequencies of CYP2C19 mutant alleles (*2, *3, and *17), genotypes, and phenotypes, and the relationship between the frequency of these alleles and the underlying hepatic diseases among patients with advanced liver diseases who were candidates for liver transplantation.

## PATIENTS AND METHODS

During the patients follow-up, 3 mL of EDTA blood samples were taken from 120 patients who were candidates for liver transplantation and who referred to the Transplant Center in Nemazee Hospital, Shiraz, Iran, from April 2014 to October 2015. Blood samples from 52 healthy individuals, the comparison group with a normal liver function, were also collected. Genomic DNAs were extracted from the leukocytes using Invisorb^®^ Spin DNA MicroKit III (Invitek, Berlin, Germany). The quantity and quality of the extracted DNA were examined spectrophotometrically (Nanodrop ND-1000, Wilmington USA). Genotyping of the extracted DNAs was performed by validated TaqMan Master Mix and TaqMan SNP genotyping assay primer and probes (C_25986767_70, C_27861809_10, and C_469857_10; Applied Biosystems, Foster City, USA). The SNPs genotypes G681A (rs4244285), G636A (rs4986893), and C806T (rs12248560) were surveyed to characterize the alleles *2, *3, and *17, respectively. Polymorphisms of CYP2C19 were determined using real-time polymerase chain reaction (ABI 7500 Fast Real-Time PCR System; Applied Biosystems, USA). The final reaction volume of 25 µL consists of 11.25 µL of template DNA, 12.5 µL of 2X TaqMan Universal Master Mix (Applied Biosystems, USA), and 1.25 µL of working stock of SNP genotyping assay, according to the manufacturer’s recommendations. The Amplification reaction was carried out as follows: an initial denaturation at 95 °C for 10 min, followed by 50 cycles of denaturation at 95 °C for 15 s, and annealing/extension at 60 °C for 90 s. All of the experiments were conducted in duplicate. CYP2C19 genotypes were classified as the phenotypes: PM, EM, HEM, and URM.

Ethics

This study was carried out in accordance with the guidelines of the Declaration of Helsinki, as revised in Edinburgh (1975). The study protocol was approved by the Ethics Committee of Prof. Alborzi Clinical Microbiology Research Center, Shiraz University of Medical Sciences, Shiraz, Iran.

Statistical Analysis

SPSS^®^ for Windows^®^ ver 18.0 was used for data analysis. To estimate the allele frequencies, data were analyzed using χ^2^ analysis and two-tailed Fisher’s exact test. The observed genotype frequencies of CYP2C19 were compared with the expected frequencies, according to the Hardy-Weinberg equation. A p value of <0.05 was considered statistically significant.

## RESULTS

This study was conducted on 120 patients (54 females, 66 males) and 52 healthy volunteers (19 females, 33 males). The mean±SD age of the participants and the comparison group was 41.7±9.0 and 35.5±11.5 years, respectively. Viral hepatitis (HBV/HCV) was the most common underlying liver disease (46/120, 38.3%), followed by primary sclerosing cholangitis (31/120, 25.8%), autoimmune hepatitis (18/120, 15%), Wilson’s disease (10/120, 8.3%), progressive familial intrahepatic cholestasis (9/120, 7.5%), and Budd-Chiari syndrome (6/120, 5%). The frequencies of CYP2C19*1 (wild type), CYP2C19*17, and CYP2C19*2 alleles were 66.7% (160/240), 20.8% (50/240), and 12.5% (30/240), respectively. No CYP2C19*3 allele was found in the studied population.

Of 120 studied patients, 57 (47.5%) had CYP2C19*1/*1 genotype, 29 (24.2%) CYP2C19*1/*17, 17 (14.2%) CYP2C19*1/*2, 9 (7.5%) CYP2C19*2/*17, 2 (1.6%) CYP2C19*2/*2, and 6 (5%) had CYP2C19*17/*17 genotypes. CYP2C19*3/*3 genotype was not expressed at all (Table 1). The prevalence rates of the CYP2C19 phenotypes for EM, HEM, URM, and PM were 47.5%, 45.9%, 1.6%, and 5%, respectively. The distribution of CYP2C19 genotype frequencies was not significantly associated with the underlying hepatic diseases (p=0.472) (Table 2).

**Table 1:** The observed and expected† genotype frequencies of CYP2C19 in patients with advanced liver diseases and the comparison group

Genotypes	Liver disease			Healthy			Anticipated Phenotypes
	Observed Number	Frequency (%)	Expected Number	Observed Number	Frequency (%)	Expected Number	
*1*1	57	47.5	51.8371	21	40.4	19.4623	Homozygous-EM
*1*17	29	24.2	33.9864	11	21.2	15.5285	Heterozygous-EM
*1*2	17	14.2	21.2954	13	25.0	13.8891	Heterozygous-EM
*2*17	9	7.5	8.2289	0	0.0	0.0	Heterozygous-EM
*2*2	2	1.6	1.1504	3	5.8	1.3883	Homozygous-PM
*17*17	6	5.0	3.5014	4	7.6	1.7316	Homozygous-URM
Overall Total	120	100	119.9996	52	100	51.9998	

PM: Poor metabolizers; EM: Extensive metabolizers; HEM: Heterozygous extensive metabolizers; URM: Ultra-rapid metabolizers

† Consistent with the Hardy-Weinberg equilibrium

**Table 2:** CYP2C19 genotype, phenotype frequencies and relationships with underlying diseases. Values are n (%

Underlying diseases	CYP2C19 Genotypes						CYP2C19 Phenotypes			
	*1*1	*1*17	*1*2	*2*17	*2*2	*17*17	EM	PM	URM	HEM
Hepatitis B, n=24	11 (46)	5 (21)	4 (17)	3 (13)	0 (0)	1 (4)	11 (46)	0 (0)	1 (4)	12 (50)
Hepatitis C, n=22	13 (59)	4 (18)	3 (14)	2 (9)	0 (0)	0 (0)	13 (59)	0 (0)	0 (0)	9 (41)
Primary sclerosing cholangitis, n=31	12 (39)	9 (29)	5 (16)	1 (3)	0 (0)	4 (13)	12 (39)	0 (0)	4 (13)	15 (48)
Autoimmune hepatitis, n=18	11 (61)	2 (11)	2 (11)	0 (0)	2 (11)	1 (6)	11 (61)	2 (11)	1 (6)	4 (22)
Wilson disease, n=10	5 (50)	3 (30)	1 (10)	1 (10)	0 (0)	0 (0)	5 (50)	0 (0)	0 (0)	5 (50)
Progressive familial intrahepatic cholestasis, n=9	4 (44)	4 (44)	1 (11)	0 (0)	0 (0)	0 (0)	4 (44)	0 (0)	0 (0)	5 (56)
Budd-Chiari syndrome, n=6	1 (17)	2 (33)	1 (17)	2 (33)	0 (0)	0 (0)	1 (17)	0 (0)	0 (0)	5 (83)
Total, n=120	57 (48.5)	29 (24.2)	17 (14.2)	9 (7.5)	2 (1.6)	6 (5.0)	57 (47.5)	2 (1.7)	6 (5.0)	55 (45.8)

Differences were not significant.

Among 52 studied healthy people, the frequencies of CYP2C19*1, CYP2C19*17, and CYP2C19*2 alleles were 63.4% (66/104), 18.3% (19/104), and 18.3% (19/104), respectively. No CYP2C19*3 allele was found in the comparison group. The frequencies of CYP2C19 genotypes were as follows: CYP2C19*1/*1 (21/52, 40.4%), CYP2C19*1/*17 (11/52, 21.2%), CYP2C19*1/*2 (13/52, 25.0%), CYP2C19*2/*2 (3/52, 5.8%), and CYP2C19*17/*17 (4/52, 7.6%) (Table 1). Extensive metabolizer, HEM, URM, and PM phenotypes were identified in 21 (40.4%), 24 (46.2%), 4 (7.6%), and 3 (5.8%) patients, respectively. The frequencies of various CYP2C19 alleles (2*, 3*, 17*) and genotypes among the comparison group were not significantly different from those in the patients. The frequencies of CYP2C19*1,*2, and *17 alleles and genotypes (not CYP2C19*3) were in Hardy-Weinberg equilibrium. 

## DISCUSSION

Having compared CYP2C19 alleles and genotypes frequencies in patients with advanced liver diseases and healthy individuals revealed no significant difference. Also, the CYP2C19 genotype frequencies were not significantly different among patients with various underlying hepatic diseases. Patients with hepatic disorders receive numerous drugs to treat their complications including both hepatic- and non-hepatic-related disorders. The best known medications are proton pump inhibitors (PPIs), antivirals, antifungals, anti-depressants, immunosuppressives, beta blockers, and pain killers. Some of these commonly prescribed drugs are metabolized by CYP2C19 [12]. Effective serum level of PPIs is needed in patients with hepatic failure, suffering from esophageal varices and gasterointestinal complications associated with drugs consumption. The lowest plasma concentration of omeprazole and 19-fold higher, were reported in URMs and PMs after similar dose of PPIs treatment by Payan, *et al* [13]. Nazir, *et al*, showed a significant rise of omeprazole serum concentration in PMs, compared with EMs indicating that lower doses of PPIs are required in PMs to reach the optimal concentrations and effects [14]. For the best management of these patients, knowledge about the CYP2C19 genotyping could be helpful to improve treatment efficacy and decreased adverse effects.

The allele frequencies of CYP2C19*1, *2, and *17 in our study were 66.7%, 12.5%, and 20.8%, respectively. Different results have been reported in other studies (Table 3). The frequencies of the CYP2C19*2 alleles found in the present study population were close to Saudi Arabian, Greek, Egyptian, and Russian populations (10.9% to 13.0%), but lower than the frequencies in the Indians and Tamilians (29.7% and 37.9%, respectively) [15-20]. CYP2C19*3 allele was absent in our study and other different populations such as Saudi Arabian, Greek, Indian, and Italian people [15, 16, 19, 21]. Its frequency was reported to be 1% and 1.8% in Iran [22, 23], 12.8% in Egypt, and 2.2% in Tamil [17, 20]. The most frequently identified variant allele in the present study was *17 (20.8%). The available data on this allele are limited, but like other alleles, its distribution is different in each ethnic group. The CYP2C19*17 frequency in Saudi Arabia was reported to be 25.7% [15], in line with our results. However, it was 1.3% in Japanese [24], which is lower than our findings. 

**Table 3:** Allele and genotype frequencies of CYP2C19 in different populations

Population	n	Reference	Allele frequency (%)				Genotype Frequency (%)								
			*1	*2	*3	*17	*1/*1	*1/*2	*1/*3	*2/*2	*2/*3	*3/*3	*1/*17	*17/*17	*2/*17
Present study	120	—	66.7	12.5	0	20.8	47.5	14.2	0	1.6	0	0	24.2	5.0	7.5
Iran	180	13	65.3	13.1	0	21.6	41.7	18.3	0	2.2	0	0	28.8	5.5	3.3
Iran	147	23	83.7	13	1	—	74.1	24.49	0.68	—	0.68	0	—	—	—
Saudi Arabia	201	15	62.9	11.2	0	25.7	40.3	14.5	0	0.4	0	0	30.4	7.0	7.0
Greece	283	16	87	13	0	—	76	21.9	0	2.2	0	0	—	—	—
Egypt	247	17	87.8	10.9	12.8	—	78.5	20	0.4	0.8	—	—	—	—	—
Russia	290	18	88.2	11.4	0.3	—	78.7	19	0.3	1.7	0.3	0	—	—	—
India	121	19	70.3	29.7	0	—	47.9	44.6	0	7.4	0	0	—	—	—
Tamil	112	20	59.8	37.9	2.2	—	29.5	58	2.7	8.0	1.8	0	—	—	—
Italy	360	21	89	11	0		79.6	18.7	0	1.7	0	0	—	—	—
Japan	265	22	57.9	27.9	12.8	1.3	35.5	43.8	—	18.8	—	—	1.1	—	—

The CYP2C19*2*2 genotype frequency, as PM in our study, was 1.6%. This result was consistent with those reported by Payan, *et al*, (1.8%) and Azarpira, *et al*, (1.3%) among Iranian healthy individuals [13, 22]. The PM genotype was reported to be 2.8% in patients with coronary artery disease [25]. The genotype frequencies of CYP2C19*2*2 among ethnicities are 2.1% in Greece, 1.7% in Russia, and 3.8% in Denmark [16, 18, 26]. The individual differences in CYP2C19 genotype status among these populations could be due to differences in the studied populations.

The distribution of frequencies of CYP2C19*1, *2, and *17 alleles in the present study followed the Hardy-Weinberg equilibrium. Being in Hardy-Weinberg equilibrium reflects a population with random mating and without immigration, mutations or natural selection (i.e., every individual has an equal chance of survival) [27]. The frequency of CYP2C19*3 was not in Hardy-Weinberg equilibrium in the present study. The small sample size of our study (one of its limitations) may lead to missed rare alleles.

The antidepressants, sertraline and escitalopram, are both metabolized by CYP2C19. Patients with defective CYP2C19 alleles (CYP2C19*2 and *3) showed higher serum levels of sertraline than those with the wild-type allele (CYP2C9*1) after administration of the same dose of the drug [28]. 

Patients with advanced liver diseases have an increased susceptibility to bacterial and fungal infections due to receiving prolonged courses of corticosteroids. The mortality rates of invasive aspergillosis in liver transplant and patients with hepatic disorders were reported 20% and 63.2%, respectively [29, 30]. Voriconazole is an effetive antifungal agent for the treatment of such infections [31, 32]. Several studies have shown the potential correlations of CYP2C19*2, *3 defective alleles with higher and CYP2C19*17 allele with lower serum voriconazole level leading to adverse events or therapeutic failure [33-35].

A wide variety of other medications including antiepileptic (phenytoin and carbamazepine), antidepressant (imipramine), antivirals (nelfinavir), and tuberculostatics (isoniazide and rifampin), extensively prescribed for patients with advanced liver diseases, are the substrate, inducer or inhibitor of CYP2C19 [36, 37]. There are potential risks of toxicity associated with use of such drugs and change in their metabolism as a result of CYP2C19 polymorphism.

In conclusion, our assessment in patients with hepatic failure and healthy individuals revealed no significant difference in the frequencies of CYP2C19 genotypes. Therefore, management of this population is the same as normal population. This may allow the physicians to predict a tailoring dose regimens based on the individual’s metabolic capacity, to decrease the risk of harmful side effects of the drugs and optimize the treatment. 
